# Genetically encoded Ca^2+^ indicators; expanded affinity range, color hue and compatibility with optogenetics

**DOI:** 10.3389/fnmol.2014.00090

**Published:** 2014-11-25

**Authors:** Takeharu Nagai, Kazuki Horikawa, Kenta Saito, Tomoki Matsuda

**Affiliations:** ^1^Department of Biomolecular Science and Engineering, The Institute of Scientific and Industrial Research, Osaka UniversityOsaka, Japan; ^2^PRESTO, Japan Science and Technology AgencyTokyo, Japan; ^3^Division for Bioimaging, Institute of Health Biosciences, The University of Tokushima Graduate SchoolTokushima, Japan; ^4^Department of Systems Neuroscience, Centre for Brain Integration Research, Tokyo Medical and Dental University Graduate School of Medical and Dental SciencesBunkyo-ku, Tokyo, Japan

**Keywords:** genetically encoded calcium ion indicator, Ca^2+^ imaging, FRET, fluorescence, bioluminescence

## Introduction

Fluorescent protein-based indicators are invaluable tools for functional imaging of living cells and organisms. Genetically encoded calcium indicators (GECIs) such as derivatives of yellow cameleons (YCs) and GCaMPs/pericams (Miyawaki et al., 1997; Nagai et al., 2001; Nakai et al., 2001) are a highly advanced class of indicators. Continued efforts for improvement of the performance of GECIs have resulted in brighter indicators with better photo-stability and expanded dynamic range, thus improving the sensitivity of detection. Fine-tuning of other properties, including Ca^2+^ affinity and Hill constant, have also contributed to increase the detectability of Ca^2+^ dynamics. Emerging optogenetic technology has forced the spectrally compatible GECI color variants. In this *opinion*, we highlight the recent development of GECIs including photo-switchable Ca^2+^ indicators and bioluminescence-based Ca^2+^ indicator, mainly invented in our group, focusing especially on the parameters determining their performance in order to provide a guideline for the selection of appropriate GECI for a given experiment.

## Affinity variant

After the first reports regarding design concept of YCs and GCaMPs/pericams (Miyawaki et al., 1997; Nagai et al., 2001; Nakai et al., 2001), their properties have been modified in term of dynamic range of signal change, pH sensitivity and color hue, and so on. However, application of these GECIs had been still limited in certain experimental targets. One of the critical limitations of these GECIs was their relatively poor repertoire of affinity variants. Because the Ca^2+^ concentration ([Ca^2+^]) at resting state and the amplitude of [Ca^2+^] change differ significantly within the subcellular locations, cell types, and organisms, a diverse set of affinity variants of GECIs covering dissociation constants (*K*_d_s) from nM to mM would be needed for studying a wide range of research targets.

While moderate- and low-affinity variants of YCs (*K*_d_ > 0.1 μM) were developed successfully by either site-directed mutagenesis of the Ca^2+^ binding domain in the indicator (Miyawaki et al., 1997) or by the rearrangement of the overall molecular structure of the indicator (Truong et al., 2001) (Table 1), there was no systematic way for engineering a high-affinity variant. *In vitro* analysis revealed that free calmodulin (CaM) and its binding peptide M13 had much higher Ca^2+^ affinity (*K*_d_ of 20 nM) than that of the CaM and M13 fusion protein linked with two amino acid linkers (*K*_d_ of 80 nM) (Porumb et al., 1994). This suggested that steric hindrance might prevent efficient interaction of Ca^2+^-CaM with M13 in YCs. This possibility was examined by serial increment of the length of the linker from 2 to 5 amino acids. Flexible linkers with 3, 4, and 5 amino acids yielded *K*_d_s of 60 nM, 30 nM, and 15 nM, respectively. Linker elongation also worked for YC 3.60, yielding five YC variants covering *K*_d_s from 15 nM to 140 nM (Table 1). These affinity variants of YCs called YC-Nano showed increased sensitivity and could detect subtle changes in [Ca^2+^] in pyramidal neurons (Horikawa et al., 2010; Yamada et al., 2011) (Table 1), becoming an ideal toolbox to efficiently monitor the novel Ca^2+^ dynamics in cases where the concentration range of Ca^2+^ is poorly described (Table 1). Recent identification of Ca^2+^ twinkle, which is a localized Ca^2+^ transient in the fine astrocytic processes, is one of the examples (Kanemaru et al., 2014).

**Table 1:** ***In vitro* properties of color variants of single-FP GECIs, selected**.

**Indicator**	**Backbone**	**Ex/Em apo**.	**Ex/Em sat**.	***D***	***K*_d_, μM**	***n***	***τ*, ms**	**p*K*_a_apo./sat**.	**References**
YC2.60	ECFP, cp173Venus	430/480(530)	–	6.6	0.04	2.4	–	–	Nagai et al., 2004
YC3.60	ECFP, cp173Venus	430/480(530)	–	6.6	0.25|0.22/0.78	1.7|3.6/1.2	2940	–	Nagai et al., 2004|Horikawa et al., 2010
YC4.60	ECFP, cp173Venus	430/480(530)	–	4.6	0.06/14.4	1.7/0.9	–	–	Nagai et al., 2004
YC-Nano140	ECFP, cp173Venus	430/480(530)	–	14	0.14/0.75	2.0/0.9	3030	–	Horikawa et al., 2010
YC-Nano65	ECFP, cp173Venus	430/480(530)	–	14	0.06/1.4	1.6/1.8	–	–	Horikawa et al., 2010
YC-Nano50	ECFP, cp173Venus	430/480(530)	–	13.5	0.05/0.4	2.5/1.0	–	–	Horikawa et al., 2010
YC-Nano30	ECFP, cp173Venus	430/480(530)	–	13.5	0.03/0.2	2.4/1.3	–	–	Horikawa et al., 2010
YC-Nano15	ECFP, cp173Venus	430/480(530)	–	15.5	0.016/0.3	3.1/0.6	–	–	Horikawa et al., 2010
D2cpV	ECFP, cp173Venus	430/480(535)	–	5.3	0.03/3.0	–	–	–	Palmer et al., 2006
D3cpV	ECFP, cp173Venus	430/480(535)	–	5.1	0.6	–	–	–	Palmer et al., 2006
D4cpV	ECFP, cp173Venus	430/480(535)	–	3.8	64	–	–	–	Palmer et al., 2006
TN-L15	ECFP, Citrine	430/480(535)	–	2.4	1.2	1	1330	–	Heim and Griesbeck, 2004
TN-XL	ECFP, cp174Citrine	430/480(535)	–	5	2.5	1.7	240	–	Mank et al., 2006
TN-XXL	ECFP, cp174Citrine	430/480(535)	–	3.3	0.8	1.5	620	–	Mank et al., 2008
PA-TNXL	PA-GFP, cp173DimVenus	504/517	–	0.18	0.22	1.4	–	–	Matsuda et al., 2013
GFP-Aequorin	Aequorin, EGFP	–/510	–	–	–	–	–	–	Baubet et al., 2000
BRAC	Venus, RLuc8	–/480(530)	–	0.6	1.9	1.3	210	–	Saito et al., 2010
Nano-lantern (Ca^2+^), CaM-2G	Venus, RLuc8_S257G	–/530	–	2	0.08/0.31	3.5/1.4	–	–	Saito et al., 2012
Nano-lantern (Ca^2+^), CaM-2GS	Venus, RLuc8_S257G	–/530	–	2	0.054/0.29	3.5/1.6	–	–	Saito et al., 2012
Nano-lantern (Ca^2+^), CaM-4GS	Venus, RLuc8_S257G	–/530	–	2	0.017/0.354	1.9/1.7	–	–	Saito et al., 2012
Nano-lantern (Ca^2+^), CaM(E104Q)-2G	Venus, RLuc8_S257G	–/530	–	2	0.62	1.4	–	–	Saito et al., 2012
Nano-lantern (Ca^2+^), CaM(E104Q)-3GS	Venus, RLuc8_S257G	–/530	–	2	0.32	1.4	–	–	Saito et al., 2012
Nano-lantern (Ca^2+^), CaM(E104Q)-4GS	Venus, RLuc8_S257G	–/530	–	2	0.065/0.321	2.5/0.7	–	–	Saito et al., 2012
G-GECO1	GCaMP3	496^*^/512	496^*^/512	26	0.75	3	700	10.0/7.6	Zhao et al., 2011
G-GECO1.1	GCaMP3 x mApple	496^*^/512	496^*^/512	27	0.62	2	700	10.0/7.5	Zhao et al., 2011
G-GECO1.2	GCaMP3 x f-pericam x mApple	498^*^/513	498^*^/513	24	1.15	2.1	700	10.0/7.2	Zhao et al., 2011
B-GECO	GCaMP3 x f-pericam x mApple	378^*^/446	378^*^/446	8	0.16	2.6	490	10.0/5.6	Zhao et al., 2011
R-GECO	cp146mApple	577^*^/600	561^*^/589	17	0.48	2	752|173	10.0/6.6	Zhao et al., 2011|Akerboom et al., 2013
GEX-GECO1	GCaMP3 x f-pericam x mApple	397^*^/512	390^*^/506	27	0.32	2.8	1030	6.0	Zhao et al., 2011
GEM-GECO1	GCaMP3 x f-pericam x mApple	397^*^/511	390^*^/455	111	0.34	2.9	225	6.2	Zhao et al., 2011
R-CaMP1.07	R-GECO	–	562^*^/ 584	28.7	~ 0.15	–	920	–	Ohkura et al., 2012
BCaMP1c	cpBFP	–	–	2	0.5	2.7	–	5.1/4.2	Akerboom et al., 2013
CyCaMP1b	cpCFP	–	–	2.6	0.42	1.7	–	7.1/8.9	Akerboom et al., 2013
YCaMP1b	cpYFP	–	–	9.2	0.8	1.7	–	7.1/8.9	Akerboom et al., 2013
RCaMP1h	cp159mRuby	575^*^/602	571^*^/594	10.5	1.3	2.5	410	–/4.9	Akerboom et al., 2013
GR-GECO1.1	cpmMaple145								Hoi et al., 2013
(Green)		487/508	491/506	3.4	0.086	2.0	1866	9.0/8.5	
(red)		559/582	564/583	3.2	0.054	2.6	1718	8.3/7.8	
GR-GECO1.2	cpmMaple145								Hoi et al., 2013
(Green)		488/506	488/506	2.2	0.074	1.7	–	9.2/8.8	
(red)		558/581	558/582	4.6	0.090	1.7	–	8.4/7.5	
	Backbone	Ex/Em apo.	Ex/Em sat.	*D*	*K*_d_, μM	*n*	τ, ms	p*K*_a_apo./sat.	references

*Ex/Em, excitation and emission wavelength; apo/sat, Ca^2+^-free/saturated condition; D, dynamic range; K_d_, dissociation constant; n, Hill constant; τ, time constant of the dissociation reaction at 20–25°C, pK_a_, acidity constant at room temperature (20–25°C). Dissociation time constant is given by τ = Θ/k_off_, assuming Θ = 1*.

* *for Ex is a peak absorption. Values from different references are separated by vertical bar. Modified from Pérez Koldenkova and Nagai (2013)*.

So far, high-affinity variants of the GCaMP and pericam families are not available (smallest *K*_d_ of 160 nM for B-GECO) (Zhao et al., 2011) (Table 1). Although these single FP-based Ca^2+^ indicators have distinct structural design unlike YCs, it will be interesting to examine whether elongation of linkers, which connect the sensor modules, contributes to increased Ca^2+^ affinity, as in the case of YC-Nano.

## Possible side effects of GECI

One might worry that the strong Ca^2+^ chelating effect of YC-Nano would affect endogenous Ca^2+^ homeostasis. Depletion of target molecules or ions by loaded indicators is often problematic for imaging of non-buffered signaling molecules, such as cyclic nucleotides and NO, but this is not the case for Ca^2+^. Like H^+^, cytosolic free [Ca^2+^] is maintained dynamically through the balancing action of Ca^2+^ buffers (i.e., Ca^2+^ binding proteins), which exist in abundance within the cell. Of course, excessive loading of Ca^2+^ indicator/chelator beyond the buffering capacity of these buffers does affect cellular Ca^2+^ homeostasis. In cases where more than mM concentrations of EGTA were loaded to observe subcellularly localized Ca^2+^, Ca^2+^ puffs and blips were generated (Cheng and Lederer, 2008). Moderate loading/expression at sub-μM concentration of YC-Nano never affected the viability of fish embryos including a set of neurons (Horikawa et al., 2010).

On the other hand, functional interference of GECIs with endogenous Ca^2+^ binding proteins and their targets could pose a problem. The CaM of YCs potentially trans-activate endogenous CaM targets. *In vitro* analysis reported that excessive amounts of CaM affect the dynamic range of conventional YCs in a dose-dependent manner (Palmer et al., 2006). To avoid these side effects, computational re-design of Ca^2+^ sensing motifs was performed. Modified binding interface of the synthetic CaM and its target prevented intermolecular interaction. The resulting YCs, named D2/3/4cpV, have been demonstrated to be insensitive to large excesses of CaM, while maintaining a *K*_d_ of 0.03–64 μM and a large dynamic range of 3.8- to 5.3-fold (Palmer et al., 2006) (Table 1).

An alternate way to avoid uncontrolled interaction of GECI with endogenous proteins is to employ a different Ca^2+^-binding motif. While CaM has a variety of downstream targets, troponin C (TnC), a skeletal and cardiac muscle-specific Ca^2+^-binding protein, is known to limit its interaction to just troponin I and troponin T. Indicators incorporating TnC from avian skeletal muscle or human cardiac muscle were generated, based on molecular design similar to that of cameleon. The resulting TN-L15 and TN-hTnC displayed a moderate Ca^2+^ affinity but a lower Ca^2+^ specificity (due to its cross reactivity with Mg^2+^) and a small signal change (due to the lack of its binding peptide which enhances the conformational change of sensor motif) (Heim and Griesbeck, 2004) (Table 1). As with YCs, further improvements have been introduced to TN-L15. Mg^2+^ reactivity was eliminated by site directed mutagenesis on TnC, and the dynamic range was increased by replacing the Citrine acceptor with its cp174 variant, eventually yielding TN-XL (Mank et al., 2006). Low affinity of TN-XL was improved in TN-XXL by replacing TnC moiety with a concatenate of its high affinity C-lobe (Table 1). Although the *in vitro* dynamic range of TN-XXL was small, its *in vivo* performance was acceptable, suggesting the advantages of using TnC with reduced interference (Mank et al., 2008) (Table 1).

## Photoactivatable GECI

To visualize Ca^2+^ dynamics in specific cell types, tissues, or organs, targeted expression of GECI gene is imperative. Although many promoters for cell/tissue/organ-specific expression are available, they do not cover all types of cell/tissue/organ. Photoswitchable GECIs (PS-GECI) can help overcome this limitation. Fluorescence status of PS-GECIs can be switched through light irradiation in arbitrary cell/tissue/organ, enabling cell/tissue/organ-specific visualization of Ca^2+^ dynamics. This “highlighted Ca^2+^ imaging” is beneficial in elucidating the activity of a single cell in the convoluted cell population of the neuronal network. There are only two reported PS-GECIs: a photoactivatable GECI, PA-TNXL (Matsuda et al., 2013), and a photoconvertible GECI, GR-GECO (Hoi et al., 2013) (Supplementary Image 1). The PA-TNXL was developed by replacing the donor and the acceptor fluorescent proteins in the TN-XL with a PA-GFP (photoactivatable GFP) and a dim yellow fluorescent protein DimVenus, respectively (Supplementary Image 1A and Table 1). Fluorescence of PA-TNXL can be switched on by violet light (~ 400 nm) irradiation. The fluorescence of the photoactivated PA-TNXL dims upon Ca^2+^ binding. GR-GECO has a similar design as GCaMPs/pericams. It has an mMaple, which can change fluorescence wavelength from green to red on being irradiated with violet light (~ 400 nm) (McEvoy et al., 2012; Hoi et al., 2013) (Supplementary Image 1B and Table 1). The intensity of both green and red fluorescence gets brighter with increase in [Ca^2+^]. For a wider range of applications, new PS-GECIs showing larger change in fluorescence intensity on photostimulation, higher dynamic range, broader Ca^2+^ affinity range, and/or reversible photoswitching are required.

## GECIs for optogenetics

In neuroscience, a paradigm shift has been brought about by optogenetics. Channel rhodopsin (ChR), a light-gated ion channel, and halorhodopsin (HR), a pump, allow us to control the activity of neural circuits with fine spatio-temporal resolution (Boyden et al., 2005; Zhang et al., 2007). As ChR and HR are activated by blue (400–500 nm) and yellow (500–600 nm) light, respectively; spectrally separated GECIs are necessary for combinatorial application of optogenetics with Ca^2+^ imaging. GECOs are the first reported color variants of single-FP GECIs based on cpGFPs and cpmApple (Zhao et al., 2011). Large-scale screening carried out by utilizing bacterial periplasmic expression system helped identify blue and red color variants of GECOs, including green and ratio metric alternates. In addition to the expanded color pallet, GECOs are also show sizable signal change (111-fold for GEM-GECO1), sensitizing them for the detection of subtle Ca^2+^ response. Structure guided evolution of GCaMP yielded BCaMP1c, CyCaM1a, YCaMP1b, and a series of RCaMPs (Akerboom et al., 2013). Compatibility of GECI color variants with optogenetic control was demonstrated by using CA3 pyramidal neuron co-expressing ChR2 and RCaMP1.07, which was in turn developed by the site-directed mutagenesis of R-GECO1 (Ohkura et al., 2012).

Bioluminescence-based Ca^2+^ imaging is an alternate and ideal strategy that is highly compatible with optogenetics. As bioluminescent indicators do not require excitation with light, observation can be free from functional crosstalk between optogenetic actuators. The limitation of this indicator was their dim signal as in the case of Ca^2+^ sensitive Aequorin and its emission-enhanced variants such as GA (Baubet et al., 2000). A considerable increase of emission signal was, however, achieved in BRAC and Nano-lantern (Ca^2+^), the latest version of bioluminescence-based GECIs (Saito et al., 2010, 2012) (Supplementary Image 1C and Table 1). BRAC is the cameleon like fluorescence resonance energy transfer (FRET)-based indicator harboring CaM-M13 moiety fused with an improved luciferase (RLuc8) derived from *Renilla reniformis*, which acts as a donor, and Venus, which acts as an acceptor. BRAC displays Ca^2+^ dependent FRET emission change (Supplementary Image 1C, left). Using BRAC, Ca^2+^ signaling was successfully visualized in plant leaves, in which fluorescence-based Ca^2+^ indicators cannot be applied due to their strong auto-fluorescence and intrinsic photosensitivity.

Nano-lantern (Ca^2+^) was constructed by direct fusion of Venus with RLuc8, which is split by insertion of CaM-M13. Ca^2+^ binding to CaM-M13 induces reconstitution of the split Rluc8. This leads to FRET from reconstituted Rluc to Venus, resulting in a large increase in emission signal (Supplementary Image 1C, right and Table 1). Thus, Ca^2+^ dynamics is monitored as a total intensity change, from both RLuc8 and FRET-enhanced emission of Venus. In the state-of-the-art demonstration by using cultured hippocampal neurons, Ca^2+^, transiently triggered by photo-activated ChR2, were imaged at 10 Hz with high SNR, showing the good compatibility of bioluminescence imaging with optogenetics (Saito et al., 2012) (Supplementary Images 1D,E).

## Conclusion and perspective

GECIs are advantageous over synthetic Ca^2+^ dyes in their targetability and reliability for chronic imaging. However, there remains room for further improvement on several parameters. Suboptimal kinetic property, non-linearity due to cooperativity, and pH sensitivity of single-FP based indicator should be improved to perform reliable detection of Ca^2+^. The future development of GECI is, regardless of the faults, promising, because of its evolvability. As a result of the past and the current attempts, not only have the basic property of GECIs been optimized, but also of new family of GECIs have been successfully developed, including color variants and self-illuminating GECIs. Future experiments will focus on improving the compatibility of GECIs with optogenetic tools. GECIs, used in collaboration with latest imaging platform such as a deep tissue imaging and ultra-fast and large-scale recording systems, will pave way to deepening our understanding of the supple ability our brain to learn and to memorize.

### Conflict of interest statement

The authors declare that the research was conducted in the absence of any commercial or financial relationships that could be construed as a potential conflict of interest.

## Supplementary material

Supplementary Image S1 **Schematic representation of photoactivatable, photoconvertible or bioluminescent Ca^2+^ indicators. (A)** PA-TNXL is composed of a troponin C (TnC) linked to a photoactivatable GFP (PA-GFP) at the N-terminal and a dim variant of Venus (DimVenus) at the C-terminal. On violet light irradiation, TnC starts fluorescing green. As the Ca^2+^ concentration increases, the intensity of fluorescence decreases proportionately due to FRET from PA-GFP to DimVenus **(B)** GR-GECO was developed from a photoconvertible FP mMaple, whose fluorescence changes from green to red on violet light irradiation. Circular permutation was introduced into mMaple and then linked to calmodulin (CaM) and M13 to create GR-GECO. The fluorescence in both green and red state increases proportionally with an increase in Ca^2+^ concentration. **(C)** A schematic representation of bioluminescence-based GECIs BRAC and Nano-lantern (Ca^2+^) for detecting Ca^2+^: Calmodulin (CaM) and M13 are used as Ca^2+^ binding domain for both of them. (Left) BRAC has Venus and RLuc8 at N- and C- terminal of CaM-M13, respectively. It causes wavelength shift from cyan to yellow thorough FRET on Ca^2+^ biding. (Right) A split RLuc is used for Nano-lantern (Ca^2+^). Each half of RLuc is linked to the terminals of CaM-M13. Compaction of CaM-M13 by Ca^2+^ binding reconstitutes the whole structure of RLuc, and luminescence intensity increases. Luminescence from RLuc is enhanced by VenusΔ C10 located at N-terminal of the Nano-lantern (Ca^2+^). An increase in Ca^2+^ can therefore be observed as FRET results in an increase in the intensity of yellow fluorescence. **(D, E)** Ca^2+^ imaging in the rat hippocampal neuron co-expressing Nano-lantern (Ca^2+^) and ChR2. **(D)** Ratio image (*L/L_0_*) of the Nano-lantern (Ca^2+^) without (i and iii) and with (ii and iv) activation of ChR2. **(E)** Time course of the ratio change. ChR2 is activated during the time period marked in gray.
