# Supplementary figures and images for: Genetically encoded Ca2+ indicators; expanded affinity range, color hue and compatibility with optogenetics
Source: Front Mol Neurosci. 2014 Nov 25;7:90. doi: 10.3389/fnmol.2014.00090 (PMC4243560; doi:10.3389/fnmol.2014.00090)

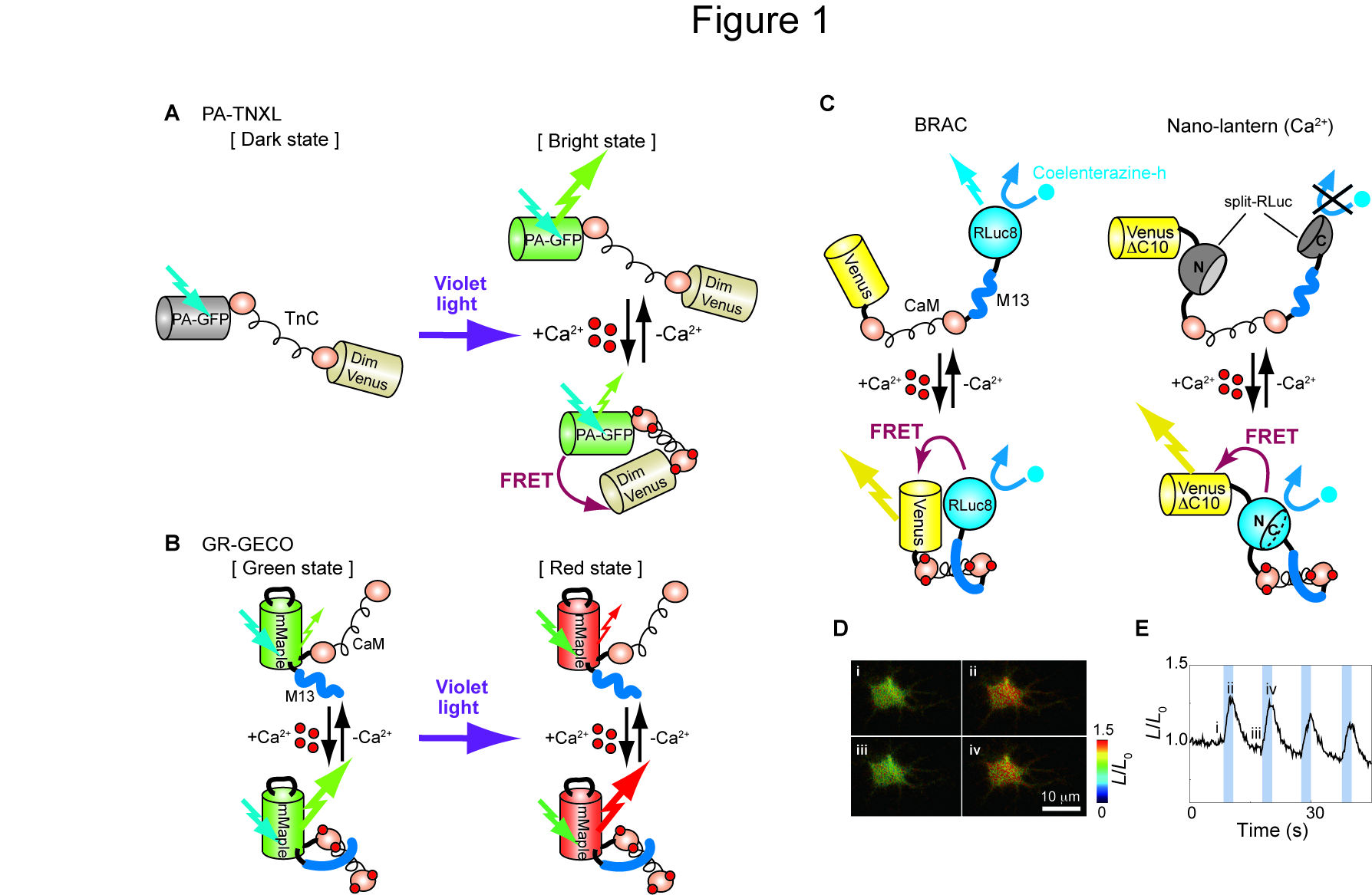

Supplement: Supplementary Image S1 — Schematic representation of photoactivatable, photoconvertible or bioluminescent Ca2+ indicators. (A) PA-TNXL is composed of a troponin C (TnC) linked to a photoactivatable GFP (PA-GFP) at the N-terminal and a dim variant of Venus (DimVenus) at the C-terminal. On violet light irradiation, TnC starts fluorescing green. As the Ca2+ concentration increases, the intensity of fluorescence decreases proportionately due to FRET from PA-GFP to DimVenus (B) GR-GECO was developed from a photoconvertible FP mMaple, whose fluorescence changes from green to red on violet light irradiation. Circular permutation was introduced into mMaple and then linked to calmodulin (CaM) and M13 to create GR-GECO. The fluorescence in both green and red state increases proportionally with an increase in Ca2+ concentration. (C) A schematic representation of bioluminescence-based GECIs BRAC and Nano-lantern (Ca2+) for detecting Ca2+: Calmodulin (CaM) and M13 are used as Ca2+ binding domain for both of them. (Left) BRAC has Venus and RLuc8 at N- and C- terminal of CaM-M13, respectively. It causes wavelength shift from cyan to yellow thorough FRET on Ca2+ biding. (Right) A split RLuc is used for Nano-lantern (Ca2+). Each half of RLuc is linked to the terminals of CaM-M13. Compaction of CaM-M13 by Ca2+ binding reconstitutes the whole structure of RLuc, and luminescence intensity increases. Luminescence from RLuc is enhanced by VenusΔ C10 located at N-terminal of the Nano-lantern (Ca2+). An increase in Ca2+ can therefore be observed as FRET results in an increase in the intensity of yellow fluorescence. (D, E) Ca2+ imaging in the rat hippocampal neuron co-expressing Nano-lantern (Ca2+) and ChR2. (D) Ratio image (L/L0) of the Nano-lantern (Ca2+) without (i and iii) and with (ii and iv) activation of ChR2. (E) Time course of the ratio change. ChR2 is activated during the time period marked in gray. [file Image1.TIF]
